# Preventing Cognitive Decline in Older Latino Adults With HIV Through a Culturally Tailored Health Promotion Intervention: Protocol for a Single-Arm Pilot Trial

**DOI:** 10.2196/55507

**Published:** 2024-08-12

**Authors:** Daniel E Jimenez, Emily J Ross, Elliott Weinstein, Hetta Gouse, Yue Pan, David Martinez Garza, Shanna L Burke, Jin Hui Joo, Victoria Behar-Zusman

**Affiliations:** 1 Department of Psychiatry and Behavioral Sciences University of Miami Miller School of Medicine Miami, FL United States; 2 Department of Surgery University of Miami Miller School of Medicine Miami, FL United States; 3 Department of Psychology University of Miami Miami, FL United States; 4 Department of Public Health Sciences University of Miami Miller School of Medicine Miami, FL United States; 5 Department of Psychiatry and Mental Health University of Cape Town Cape Town South Africa; 6 School of Social Work Robert Stempel College of Public Health & Social Work Florida International University Miami, FL United States; 7 Department of Psychiatry Massachusetts General Hospital Harvard Medical School Boston, MA United States; 8 School of Nursing and Health Studies University of Miami Miami, FL United States

**Keywords:** Latinos, HIV, AIDS, cognitive decline, health promotion, intervention, protocol, single-arm, pilot trial, prevention, older, cognitive impairment, impairment, treatment, dementia, psychosocial, men, women, cohort

## Abstract

**Background:**

Older Latino adults with HIV are at increased risk for mild cognitive impairment and earlier onset of aging-related cognitive decline. Improvements in cognitive functioning and cognitive outcomes are possible among people with HIV who adopt health promotion behaviors. However, health promotion interventions for older Latino adults with HIV have not been extensively used or widely recognized as viable treatment options. Happy Older Latinos are Active (HOLA) is a multicomponent, health promotion intervention that is uniquely tailored for older Latino adults with HIV.

**Objective:**

This study aims to (1) determine the feasibility and acceptability of an adapted version of HOLA aimed at improving cognitive functioning among older Latino adults with HIV; (2) explore whether HOLA will produce changes in cognitive functioning; (3) explore whether HOLA will produce changes in activity, psychosocial functioning, or biomarkers of cognition; and (4) explore whether changes in activity, psychosocial functioning or cognitive biomarkers correlate with changes in cognition, while accounting for genetic risk for dementia.

**Methods:**

A single-arm pilot trial with 30 Latino (aged 50 years and older) men and women with HIV was conducted to assess feasibility, acceptability, and preliminary effects on cognition. Participants were assessed at 2 time points (baseline and postintervention) on measures of neurocognitive and psychosocial functioning. In addition, blood samples were collected to determine biomarkers of cognition at baseline and postintervention. Successful recruitment was defined as meeting 100% of the targeted sample (N=30), with 20% (n=6) or less of eligible participants refusing to participate. Adequate retention was defined as 85% (n=25) or more of participants completing the postintervention assessment and acceptability was defined as 80% (n=38) or more of sessions attended by participants.

**Results:**

Participant recruitment began on February 22, 2022, and was completed on August 15, 2022. The last study visit took place on February 20, 2023. Data analysis is currently ongoing.

**Conclusions:**

Encouraging findings from this exploratory study may provide a blueprint for scaling up the HOLA intervention to a larger cohort of older Latino adults with HIV who may be currently experiencing or are at risk for HIV-related cognitive challenges.

**Trial Registration:**

ClinicalTrials.gov NCT04791709; https://clinicaltrials.gov/study/NCT04791709

**International Registered Report Identifier (IRRID):**

DERR1-10.2196/55507

## Introduction

Older Latino adults with HIV are disproportionately affected by the HIV epidemic and comorbid health challenges at increasing rates [1-4]. Although Latino adults represent approximately 19% of the US population, they account for 21% of new HIV infections, and 20% of those living with the virus. These disparities become even more pronounced among older Latino adults [4] with HIV incidence rates for Latino individuals in the United States being 5 times as high compared to their non-Latino White counterparts [1]. Additionally, Latino individuals with HIV are diagnosed later in life with approximately 17% of them receiving their diagnosis after the age of 50 [1]. These inequities already contribute to poorer HIV-related and general health outcomes among older Latino adults with HIV and will continue to magnify with the “greying of the epidemic” [5].

Older Latino adults with HIV are uniquely at risk for experiencing deleterious neuropathological and neurocognitive sequelae within the context of aging due to the co-occurrence of aging-related cognitive impairments and depression [1,2,4,6]. Although advancements in HIV treatment (eg, antiretroviral therapy [ART] over the past 2 decades have greatly improved the longevity and health of people with HIV [7], neurocognitive impairments are observed in approximately half of people with HIV [8]. Nonpathological cognitive impairments— or age-related cognitive changes—have been well-documented in numerous aging [7,9,10] and HIV-centered studies [7,11-13]. Although HIV-related dementia is uncommon in the era of highly effective ART [10,14-16], people with HIV remain susceptible to both cortical and subcortical insults that facilitate cognitive impairments [17,18]. For example, in 1555 adults with HIV from across the United States, Heaton et al [8] found that 52% of people with HIV experienced measurable cognitive impairments, with 33%, 12%, and 5% experiencing asymptomatic neurocognitive impairment, mild neurocognitive disorder, and mixed neurocognitive disorder, respectively.

Such cognitive impairments may be attributable to a diverse set of factors related to HIV pathogenesis and progression. These factors include HIV-induced death of glial cells; elevated cortisol levels and inflammation caused by HIV; age- and HIV-associated comorbidities; and ART-induced metabolic complications such as hypercholesterolemia and insulin resistance [15,17,19-23]. In a cross-sectional study of middle-aged and older veterans with HIV, Justice et al [24] found a high prevalence of cognitive impairments (eg, speed of processing, memory). Furthermore, these cognitive challenges are more acutely exacerbated among older adults with unsuppressed viral load [25] and may even present as early as 1-year post-HIV acquisition [26-29].

Older Latino adults with HIV encounter distinct challenges in cognition that stem directly from the intersection of mental health challenges (eg, depression), HIV, and general aging-related cognitive impairment. Ethnic differences have been found in speed of processing, memory, and reasoning/executive functioning—the cognitive domains most often impacted by HIV-related challenges—with older Latino adults with HIV experiencing higher rates of impairment in these aspects of cognition [4,6,10,20,30-45]. Depression, which has also been identified as an independent risk factor for cognitive impairment, has been reported at 5 times the rate among older Latino adults with HIV than in the general population [6,7,45]. The causes of depression among older Latino adults with HIV are multifaceted. Two of the most influential contributing factors may be stigma and resultant loneliness due to social rejection and withdrawing from society [6,46].

There are many social and lifestyle factors that contribute to declining cognitive reserve within the context of HIV. For example, when Fazeli et al [31] examined the relationship between lifestyle factors and the prevalence of cognitive impairment in people with HIV, they found that if participants were physically active (ie, engaged in any strenuous exercise in the past 72 hours), socially and or behaviorally activated, and mentally stimulated (ie, working full- or part-time), participants had a greatly reduced likelihood of presenting with cognitive impairment [31]. This observation parallels findings in the cognitive aging literature that supports the notion that disengaging from a sedentary lifestyle is good for both one’s physical and cognitive health [47]. Self-reported sedentary behavior is associated with greater odds of impaired learning, motor function, and memory in adults with and without HIV [48]. Moreover, people with HIV who reported regular physical activity over 2.5 years maintained better neurocognitive function even when compared with sedentary adults without HIV [49].

Physical activity is known to produce structural and cellular changes within the brain including promoting angiogenesis, synaptogenesis, neurogenesis, and dendritic remodeling [48-52]. Several theories suggest that physical activity stimulates the release of a number of factors from adipose tissue and skeletal muscles that produce these changes in the brain. Many of the molecules suspected to potentiate this process include myokines or cytokines (interleukin [IL]-15), adipokines (irisin and adiponectin), and neurotrophic factors (brain-derived neurotrophic factor [BDNF], insulin-like growth factor 1 [IGF-1], and vascular endothelial growth factor [VEGF]), which are detected in the blood [52]. Such biomarkers can be used to determine the impact of physical activity on brain health, and possibly function [52].

The salience of cognitive impairment among older Latino adults with HIV suggests that health promotion interventions—defined as behavioral interventions that use counseling strategies to equip participants with the necessary knowledge and skills to modify and sustain a healthy diet, increased physical activity, or healthy weight—are well-aligned with their needs and may provide a practical approach to address them. Health promotion is safe, and the benefits for older Latino adults with HIV who are pharmacologically burdened is well documented [45]. Similarly, improvements in both cognitive functioning and outcomes are possible among people with HIV who adopt health promotion behaviors [12]. However, despite such encouraging evidence, health promotion interventions for midlife and older Latino adults with HIV have not been extensively used or widely recognized as viable treatment options, perhaps due to the fact that the majority of older Latino adults with HIV are more likely to be sedentary and not as actively engaged in pursuing changes in their physical activity compared to their non-Latino White counterparts [53].

**Figure 1:**
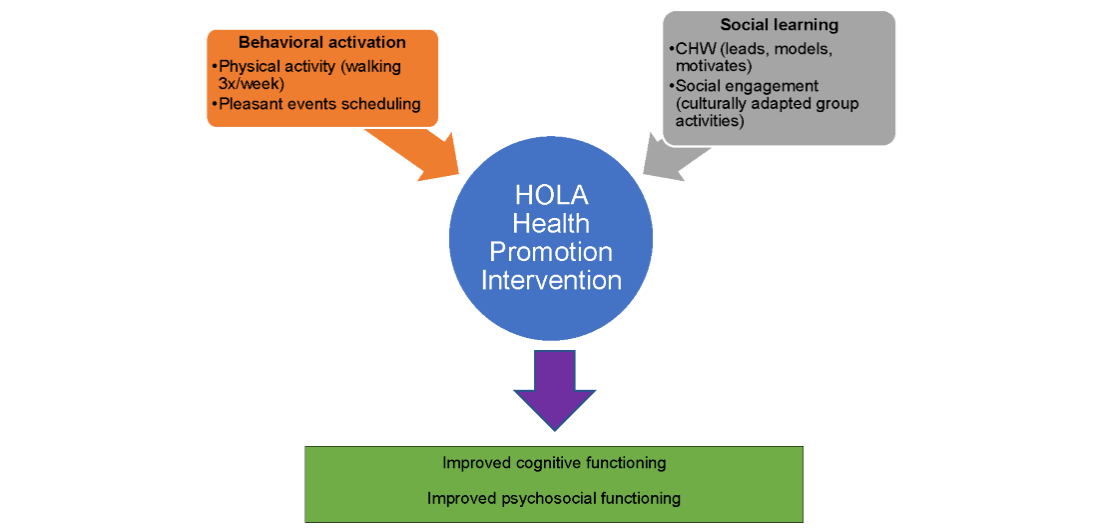
HOLA conceptual framework. CHW: community health worker; HOLA: Happy Older Latinos are Active.

Happy Older Latinos are Active (HOLA) is a multicomponent, health promotion intervention that incorporates a moderate intensity group walk, uniquely tailored for older Latino adults with HIV. Informed by behavioral activation and social learning theory [54,55], HOLA builds on prior research [56] using health promotion to prevent depression and anxiety in older Latino adults. Through physical activity and engaging in pleasant and meaningful activities, behavioral activation helps older Latino adults with HIV break the cycle of inactivity and social withdrawal to improve cognitive and psychosocial functioning. To complement this process, we use the constructs of observational learning, reinforcement, and enhanced self-efficacy postulated in social learning theory [55]. The personal relationship between the participants and the community health worker (CHW) is an important factor that helps to motivate, model, and maintain health behavior change. The CHW holds the individuals accountable, and individuals hold themselves accountable to the group, providing additional motivation to engage in the intervention. Figure 1 depicts the conceptual framework of HOLA.

HOLA is feasible and acceptable among older Latino adults [56]. Notably, results from a previous pilot study suggested that participants who engaged in the intervention expressed interest in learning more evidence-based methods to prevent cognitive decline, even though HOLA had not previously been piloted with a focus on cognition. To address this gap, a single-arm trial was conducted using HOLA with older Latino adults with HIV to assess feasibility, acceptability, and preliminary effects on cognition. The 4 specific aims of this single arm trial were (1) to determine feasibility and acceptability of an adapted version of HOLA aimed at improving cognitive functioning among older Latino adults with HIV and incorporating activity tracking; (2) explore whether there are cognitive differences in HOLA participants between baseline and posttest assessments; (3) explore whether participation in HOLA results in changes in activity, psychosocial functioning (depression, anxiety, and social support) or biomarkers of cognition (myokines or cytokines [IL-15], adipokines [irisin and adiponectin], neurotrophic factors [BDNF, IGF-1, and VEGF]) between baseline and posttest assessments; and (4) explore whether changes in activity, psychosocial functioning, or cognitive biomarkers correlate with changes in cognition, while accounting for genetic risk for dementia (apolipoprotein E [APOE] ε4 carrier status).

## Methods

### Study Design

In this study, 30 older Latino adults with HIV aged 50 years and older were enrolled in a single arm trial to assess the feasibility and acceptability of the HOLA health promotion intervention and to identify preliminary intervention effects on cognitive functioning. The mean age at baseline was 61.7 (SD 6.0) years, and the sample was predominantly men (60%; n=18). Participants were assessed at 2 time points (baseline and 16 weeks post intervention) on measures of neurocognitive and psychosocial functioning. Blood samples were collected to assess biomarkers of cognition. Consistent with recommendations from biostatistical workgroups funded by National Institutes of Health (NIH) [12], this pilot study was not powered to test hypotheses. Participants were recruited from the Center for HIV and Research in Mental Health which serves as a consent to contact database, to efficiently and ethically contact eligible participants who receive HIV care at the University of Miami or Jackson Health System HIV Clinic [57].

### Participants

Inclusion and exclusion criteria for study participation are outlined in Textbox 1.

Notably, the age of 50 years was chosen as a cutoff for 2 reasons. First, more than half of individuals affected by HIV in the United States are aged 50 years or older [2]. Second, in HIV infection, patients aged 50 years and older have poorer immunological response to ART and poorer survival. This age has been used to define medically advanced age in HIV-infected people [58]. Because of the longitudinal study design, participants planning to move outside of the South Florida metropolitan area within 6 months of enrollment or not living in stable housing (eg, group home) were excluded. All participants read and signed an informed consent form approved by the University of Miami institutional review board (IRB).

**Criteria for Happy Older Latinos are Active (HOLA) study participation.**

**Inclusion criteria**

- Self-identify as Latino
- Aged 50 years or older
- Been diagnosed with HIV
- Maintained virological suppression (viral load <200 copies/mL)
- Volunteer informed consent
- Have medical clearance by a physician to participate in a physical activity intervention.

**Exclusion criteria**

- Have a diagnosis of any neurodegenerative disorder or dementia (Parkinson disease, Alzheimer disease, vascular, frontotemporal dementia) or significant cognitive impairment as indicated by a Telephone Interview for Cognitive Status score <30.
- Have other conditions that could impact cognitive functioning or testing (eg, legally blind or deaf)
- Currently undergoing radiation or chemotherapy.
- History of brain trauma with a loss of consciousness greater than 30 minutes.
- Contraindications to physical activity outlined in the American College of Sports Medicine standards or severe medical illness that precludes them from safely participating in a health promotion intervention.
- Unable to complete 10-meter walk test.

### Measures and Assessment Protocols

Table 1 describes the measurement battery and assessment schedule.

The neurocognitive battery represents the domains that are used to classify cognitive impairment. These cognitive performance tests are norm-based, allowing us to detect performance comparisons to one’s age and education level. Multiple equivalent versions of these tests are available which reduces the potential confound of practice effects. Study measures were administered in either English or Spanish (using certified translations), according to the participant’s preference. Trained bilingual research assistants (RAs) conducted all assessments in private offices at the University of Miami or Jackson Health System HIV Clinic. A licensed neuropsychologist trained and supervised the RAs using a multistep process. First, the training focused on understanding test administration procedures, scoring guidelines, and potential difficulties of the specific tests that were to be administered. Second, the RAs observed the neuropsychologist administer the assessment battery. The RAs then practiced administering the test under direct supervision before transitioning to conducting the assessments on their own. The neuropsychologist would conduct interreliability checks and provide regular feedback and address any errors.

**Table 1:** Measurement battery.

Criteria		Instrument
**Screening and background variables (administered at baseline only)**		
	Cognitive screen	TICS^a^ [59]
	Walking ability	10-meter walk test [60]
	Demographics	CLaRO^b^ demographics form
	Acculturation	Bidimensional acculturation scale [61]
**Psychosocial functioning (administered at baseline and 16 weeks)**		
	Depression severity	PHQ-9^c^ [62]
	Anxiety severity	GAD-7^d^ [63]
	Perceived social support	Multidimensional Scale of Perceived Social Support [64]
**Physical activity and characteristics (administered at baseline and 16 weeks)**		
	Waist and hip circumference	Direct measurement
	Physical activity	GPAQ^e^ [65]; Fitbit activity tracker
**Neurocognitive functioning (administered at baseline and 16 weeks)**		
	Speed of processing	Trail making test A [66-69]
	Attention or working memory	WAIS-III^f^ spatial span [69,70]
	Learning	BVMT-R^g^ trials 1-3 [71,72]
	Memory	BVMT-R delay [71,72]
	Verbal fluency	Animal fluency [24,73]
	Executive function	Trail making test B [66-69,73]
	Motor	Grooved pegboard (dominant and nondominant) [74]
**Participant feedback (administered at 16 weeks)**		
	Participant satisfaction	CSQ-8^h^ [75]

^a^TICS: Telephone Interview for Cognitive Status.

^b^CLaRO: Center for Latino Health Research Opportunities.

^c^PHQ-9: Patient Health Questionnaire-9.

^d^GAD-7: Generalized Anxiety Disorder-7 Scale.

^e^GPAQ: Global Physical Activity Questionnaire.

^f^WAIS-III: Wechsler Adult Intelligence Scale-III.

^g^BVMT-R: Brief Visuospatial Memory Test, Revised.

^h^CSQ-8: Client Satisfaction Questionnaire-8.

At the end of the intervention, participants’ satisfaction with the intervention was measured using the 8-item Client Satisfaction Questionnaire-8 [75]. At the end of the Client Satisfaction Questionnaire-8, there are 2 open-ended questions that allow participants to state in their own words what they liked most about the intervention and what they liked the least. Answers to these questions will be used to identify modifications needed in the design of a larger, ensuing hypothesis testing study.

### Biological Data Protocol

Blood draws took place at the University of Miami or Jackson Health System HIV Clinic prior to starting baseline assessments. Approximately 20 mL (2 tablespoons) of blood was collected by a trained phlebotomist at both time points to assay biomarkers of cognition: myokines or cytokines (IL-15), adipokines (irisin and adiponectin), and neurotrophic factors (BDNF, IGF-1, and VEGF). APOE status, a genetic marker of dementia risk, was collected at baseline only. The biomarkers were assayed at the University of Miami Center for AIDS Research Laboratory Sciences Core using a commercially available enzyme-linked immunosorbent assay.

### Description of the HOLA Intervention

HOLA is a multicomponent health promotion intervention for older Latino adults. The first component consists of 2 manualized social and physical activation sessions. Prior to beginning the group walk phase, each participant met individually with a CHW for a 30-minute physical and social activation session to (1) educate participants about the goals of the intervention; (2) provide information surrounding cognitive functioning, age-related memory loss versus dementia, how their comorbidities impact cognition, and ways they can prevent cognitive decline; (3) motivate participants to engage in physical activity; (4) increase participants’ social activities; (5) identify potential obstacles that may interfere with meeting the demands of the intervention; and (6) brainstorm ways to overcome these obstacles. Participants met again individually with the CHW for 30 minutes after week 8 to discuss progress of physical and social activity goals. These meetings were typically held in the participants’ homes.

HOLA’s second component is a group walk, led by a CHW for 45 minutes, 3 times a week, for 16 weeks. Walks were conducted with a group of 6 participants at a centrally located public park. Each walk began with 10 minutes of stretching and warm up and followed by 25 minutes of moderately intense walking. The group walk concluded with a 10-minute cooldown phase of stretching.

The third component consists of scheduling pleasant events, which offers a means to generalize the intervention into the participants’ everyday lives and relationships. During the cool-down phase of each walking session, the CHW asks each participant to identify a pleasant event that they intended to do with another person before the next meeting. Subsequent sessions start with participants reporting on how effectively they implemented their pleasant event plan while the CHW and the group provided positive reinforcement and feedback.

Cultural values and beliefs were included in the formative phases of HOLA to ensure that it addressed sociocultural influences that have a role in the health of older Latino adults. First, recruitment, intervention, and assessment materials were available in English and Spanish, and we added further cultural components to the social and physical activation manual by including Latino relevant metaphors, values, and cultural proverbs (dichos). Second, our recruitment strategies focused on relationship building given the fear, mistrust, and stigmatizing attitudes in the Latino community toward participation in clinical research. Specifically, we emphasized the Latino cultural values of personalismo (the cultural value that emphasizes social relationships and the mutual respect that those in the relationship have for one another) and confianza (trust and familiarity). Third is the use of CHWs. Since CHWs possess an intimate understanding of the community, they understand cultural values and norms and know how to incorporate them in order to promote health and health outcomes. Moreover, older Latino adults with HIV may feel more comfortable divulging personal information to someone from their own community, allowing them to communicate with study personnel in their preferred language and in a manner in which they feel comfortable.

### CHW Training and Supervision

The CHW in this study worked on a previous HOLA trial [76] and was already trained to deliver the HOLA intervention using a detailed manual of operations. A clinical psychologist met weekly with the CHW to provide supervision and give one-on-one corrective feedback as needed to ensure that what was being delivered was fully consistent with the intervention as designed. Furthermore, all planned and unplanned contacts were recorded. This involved the CHW documenting the following variables for each contact with a participant: date, duration, type of session, initiator of contact, planned or unplanned contact, reason for contact, and activities engaged in.

### Data Analysis

For this pilot study, we evaluated the feasibility of recruitment, assessment procedures, retention, acceptability, and implementation of HOLA in a sample of older Latino adults with HIV (aim 1). Consistent with recommendations from biostatistical workgroups funded by NIH [39], this pilot study was not powered to test a hypothesis; therefore, we did not complete a power analysis. Successful recruitment was defined as meeting 100% of the targeted sample (N=30), with 20% (n=6) or less of eligible participants refusing to participate. Adequate retention was defined as 85% (n=25) or more of randomized participants completing the postintervention assessment, and acceptability was defined as 80% (n=38) or more of sessions attended by participants.

To explore changes in neurocognitive impairment (memory, reasoning or executive functioning, speed of processing; aim 2), and general activity, psychosocial functioning, and biomarkers of cognition (aim 3), a 1-way ANOVA will be used to illustrate the change in scores from baseline to postintervention in all the outcome measures. General linear modeling will be used to explore whether changes in psychosocial functioning or cognitive biomarkers (myokines or cytokines [IL-15], adipokines [irisin and adiponectin] and neurotrophic factors [BDNF, IGF-1, and VEGF]) correlate with changes in cognition, while accounting for genetic risk for dementia (APOE ε4 carrier status). Due to the small sample size, APOE genotypes will be collapsed into ε4 carrier status carriers and non-ε4 carriers (aim 4).

Given that depression and cognitive functioning are closely linked, it is possible that improvements in cognitive functioning could be due to reductions in depressive symptoms. Therefore, to understand better the intervention effect on cognitive functioning, we will use generalized estimating equation with a log link function and Poisson distribution. The model will estimate the incident rate ratio for cognitive functioning outcomes at follow-up versus baseline. All generalized estimating equation models will include time as the main effect and control for gender, age, socioeconomic status, years of education, and depression severity at baseline as covariates. In addition, a mediational analysis will be done using structural equation models to determine whether intervention effects, if detected, are influenced by depression.

### Ethical Considerations

Approval from the University of Miami IRB was obtained on May 14, 2021 (IRB ID 20201202). In addition, the trial was registered in the ClinicalTrials.gov database on March 5, 2021 (NCT04791709). All study procedures were conducted according to good clinical practice and all other relevant regulatory guidelines. Participants were informed that they may end their participation in the study at any time without affecting access to any other services they were receiving. Participant payments were graduated such that participants received US $25 on the baseline visit and US $35 on the follow-up visit (total honoraria: US $60). They also got to keep the tracking device.

Completed data were entered into password secured databases by authorized staff. All electronic data were deidentified and stored in password-protected files on servers at the University of Miami Miller School of Medicine Department of Psychiatry and Behavioral Sciences. For each data file, a code number was assigned, and the master list, linking code numbers with names, was stored separately. At the conclusion of the project, the record linking the assigned research number and participant identity will be destroyed. No participant will be identified within any published report. Each member of the research team was certified and trained in maintenance of confidentiality and data compliance standards.

## Results

Participant recruitment began on February 22, 2022, and was completed on August 15, 2022. The study was completed on February 20, 2023. Data analysis is currently ongoing with a goal of completion by late December 2023 and a paper submission during the third quarter of 2024.

## Discussion

### Expected Findings

To the best of our knowledge, this is the first study to adapt a culturally tailored health promotion intervention for older Latino adults with HIV to improve physical, mental, and cognitive health. Findings from this pilot study will help to identify whether the HOLA health promotion intervention is (1) feasible and acceptable to a group of older Latino adults with HIV, and (2) potentially efficacious in bolstering brain health in this underserved group. Encouraging findings from this exploratory study may provide a blueprint for scaling up HOLA to a larger cohort of older Latino adults with HIV who may be currently experiencing or at risk for HIV-related cognitive challenges.

There are several benefits to this single arm pilot trial. At the individual level, older Latino adults with HIV may acquire and practice health behaviors that lead to improved well-being by participating in this intervention. Similarly, engaging with this intervention offers older Latino adults with HIV the opportunity to receive support, advice, encouragement, and feedback from a trained CHW and other participants who are peers with similar lived experiences as themselves. Continued psychoeducation on physical, mental, and cognitive health from the CHW as well as emotional, instrumental, informational, and appraisal support from peers may help participants gain enhanced motivation to take control of their health while also increasing psychological well-being and self-efficacy.

This project has direct public health implications at the societal level. Findings from this research may offer insight into how a health promotion intervention could improve cognitive functioning among older Latino adults with HIV with implications for dementia prevention. Similarly, older Latino adults, particularly those with HIV, are at increased risk for developing depression and anxiety compared to their White age-matched peers. Therefore, based on the intervention’s focus on facilitating activity and increasing social support among older Latino adults with HIV, HOLA may chip away at social isolation among participants through opportunities for socialization and relationship-building with peers, leading to general mental health improvements in the long-term. In addition to improving the general well-being of older Latino adults with HIV, the development of more efficacious interventions that stave off cognitive and mental health challenges prior to their onset among this group is cost-effective and reduces future health care burden costs. Ultimately, approaches such as the HOLA intervention shift the attention toward preventing morbidity rather than treating it.

Finally, this study is highly relevant and responsive to the NIH priority to “maintain or improve the cognitive performance of all adults,” especially for those “populations experiencing the greatest disparities and risks in cognitive health” [77]. This project shares the same values as both the NIH Cognitive and Emotional Health Project and the Healthy Brain Initiative (a national road map to maintaining cognitive health) and offers a noninvasive technique to improve cognition in adults with HIV, a major challenge currently facing the field [17].

### Limitations

This study has some limitations. First, it targeted older Latino adults with HIV living in the southeastern United States; therefore, study findings may not be generalizable to older Latino adults with HIV living in other parts of the United States or globally. Second, participants were older Latino adults with HIV who were engaged in HIV care and living who have a suppressed HIV viral load. In order to support older Latino adults with an unsuppressed HIV viral load, this intervention may need to be tailored to accommodate them. Third, participants who were not living in stable housing were excluded from the study. Including participants who were not living in stable housing—which typically make up a fairly large portion of people living with HIV—would have provided interesting and useful information about whether they would have been able to attend the intervention and how they would have fared in the study. However, given that we only had 1 year to recruit 30 participants and run them through a 16-week intervention, we had to make the most efficient use of our time. Therefore, the decision was made to only include participants who were stably housed. We intend to remove this criterion in a subsequent, adequately powered trial. Fourth, the study was not powered to test for intervention effects. However, any effects that are found to be statistically significant would be encouraging. Nonetheless, this single-arm pilot study used a sound methodological approach, and, if successful, data gleaned will advance the field of HIV-related cognitive impairment among older Latino adults with HIV.

## Abbreviations

APOE: apolipoprotein E
ART: antiretroviral therapy
BDNF: brain-derived neurotrophic factor
CHW: community health worker
HOLA: Happy Older Latinos are Active
IGF-1: insulin-like growth factor 1
IL: interleukin
IRB: institutional review board
NIH: National Institutes of Health
RA: research assistant
VEGF: vascular endothelial growth factor

## Data Availability

The data sets generated during and analyzed during this study are available from the corresponding author on reasonable request.
